# Post-concussive Dizziness: A Review and Clinical Approach to the Patient

**DOI:** 10.3389/fneur.2021.718318

**Published:** 2022-01-04

**Authors:** Gerard J. Gianoli

**Affiliations:** The Ear and Balance Institute, Covington, LA, United States

**Keywords:** post-concussion dizziness, post-concussion syndrome, dizziness, concussion, treatment, management, vertigo, traumatic brain injury

## Abstract

Dizziness is a frequent complaint after head trauma. Among patients who suffer a concussion (mild traumatic brain injury or mTBI), dizziness is second only to headache in symptom frequency. The differential diagnosis of post-concussive dizziness (PCD) can be divided into non-vestibular, central vestibular and peripheral vestibular causes with growing recognition that patients frequently exhibit both central and peripheral findings on vestibular testing. Symptoms that traditionally have been ascribed to central vestibular dysfunction may be due to peripheral dysfunction. Further, our ability to test peripheral vestibular function has improved and has allowed us to identify peripheral disorders that in the past would have remained unnoticed. The importance of the identification of the peripheral component in PCD lies in our ability to remedy the peripheral vestibular component to a much greater extent than the central component. Unfortunately, many patients are not adequately evaluated for vestibular disorders until long after the onset of their symptoms. Among the diagnoses seen as causes for PCD are (1) Central vestibular disorders, (2) Benign Paroxysmal Positional Vertigo (BPPV), (3) Labyrinthine dehiscence/perilymph fistula syndrome, (4) labyrinthine concussion, (5) secondary endolymphatic hydrops, (6) Temporal bone fracture, and (7) Malingering (particularly when litigation is pending). These diagnoses are not mutually exclusive and PCD patients frequently exhibit a combination of these disorders. A review of the literature and a general approach to the patient with post-concussive dizziness will be detailed as well as a review of the above-mentioned diagnostic categories.

## Introduction

Mild traumatic brain injury or concussion (mTBI) has been reported to occur more than a million times annually in the U.S. (1). That is likely an undercount since many mTBI do not present for treatment. Approximately 50% of these patients will have persistent symptoms, i.e., Post-Concussion Syndrome, at 1 month and 15% at 1 year. Dizziness is the second most common symptom of concussion (mTBI) and predictive of a prolonged recovery from Post-Concussive Syndrome. However, the symptom “Dizziness” is ill-defined at best. Definitions range from “giddiness” to unsteadiness to rotary vertigo spells. Patients often find it difficult to describe their dizziness, and even patients who ultimately are determined to have the same disorder will describe the symptoms very differently. Exactly what is meant by dizziness, however, is probably not as important as what provokes the symptom. There are multiple pathologies that can potentially provoke the symptoms of dizziness of which we will explore in this paper. While it seems obvious that damage to the brain can cause “dizziness”, damage to the inner ear is an under-recognized and quite common source for the symptom of dizziness.

## Pathophysiology

The mechanism of brain trauma from concussion is likely due to both direct and indirect factors. Direct compressive and tensile forces occur during the “coup” and “contrecoup” impacts of the brain against the inside of the skull. In addition to these compressive and tensile forces are rotational forces that result in axonal shearing. Diffuse axonal injury (DAI) presumably results in several of the symptoms seen in post-concussion syndrome. Post-trauma perfusion changes, biochemical changes and inflammation can lead to additional pathology. These events and DAI in the central vestibular system with its connections in the cerebellum and brainstem doubtless could result in symptoms of dizziness secondary to concussion (2).

Second impact syndrome (3) is a rare and devastating phenomenon which occurs when a patient has a second head injury while still recovering from mTBI. This can result in elevation of intracranial pressure, subsequent herniation, and death. It is almost exclusively seen in the young and is believed to be due to dysregulation of cerebral blood flow and resulting edema. Prevention of Second Impact Syndrome by delaying return of athletes to competition until after recovery from mTBI is imperative.

The pressure effects from mTBI will extend to the intralabyrinthine compartment and has historically been termed Labyrinthine Concussion (LC). Bartholomew and colleagues (4) recently published a historical review of the research into LC. In numerous animal studies and clinical reports dating back to the late 1800s, concussions have been reported to result in direct inner ear damage. The first pathology reported was intralabyrinthine hemorrhage. Intralabyrinthine hemorrhage has been most reported to occur in the basilar turn of the cochlea and the vestibule. More extensive hemorrhage was noted with more severe trauma. However, the presence of hemorrhage did not necessarily correlate with the location of end organ damage.

Other researchers proposed cochleovestibular nerve traction injury as the presumed mechanism for audio-vestibular dysfunction in head injured patients. This was supported by the finding of blood in the internal auditory canal. This theory, although it may be the cause in some cases of head trauma, fell out of favor as further evidence came forth.

The traveling wave theory of LC became the main theory for inner ear damage from LC in the acute phase of injury. The proposed mechanism of a pressure wave traveling through the inner ear causing disruption of the end organ neuroepithelium was greatly supported by animal research by Schuknecht (5). In a series of animal experiments, Schuknecht conditioned cats to perform behavioral audiometry. The post-injury audiograms correlated with the postmortem histologic findings. These experiments support the traveling wave theory at least in the acute phase of injury. The subacute and chronic phases of LC have been proposed to include vasomotor dysfunction, progressive inflammatory states, secondary degenerative changes, intralabyrinthine ossification, endolymphatic hydrops and synaptopathy.

## Histopathology

In human temporal bone histology, the findings of LC include pathologic changes to the cochlea and to the vestibular end organs. Reviewing cochlear damage, Ishai et al. (6) reported on 5 temporal bone specimens of patients who had head injury without temporal bone fracture and other etiologies for inner ear pathology. All specimens had moderate to severe degeneration of spiral ganglion cells. Several cases demonstrated hair cell loss. In half the cases, cochlear hydrops was noted.

Knoll et al. (7) performed a similar evaluation examining vestibular damage of 8 temporal bone specimens of patients without temporal bone fracture, excluding etiologies for other inner ear pathology. They found moderate to severe degeneration of the membranous vestibular labyrinth in half of the cases. In the 5 cases with intact specimens, moderate atrophy of the superior and inferior vestibular nerves was noted. Mild to severe otolithic maculae degeneration was noted in 25% of cases. Vestibular hydrops was present in 25% of cases and there was one case of endolymphatic duct blockage. Of note, otoconia (either on the otolithic membrane or free floating) was not able to be assessed due to the EDTA decalcification process. Consequently, no assessment could be made of BPPV or otoconial damage.

## Testing

It has long been recognized that concussions can lead to hearing loss and tinnitus. Schuknecht's above-mentioned study with cats confirmed the most common hearing loss to occur was a sensorineural hearing loss with notching at 3–8 kHz—quite similar to what is seen with occupational noise-induced hearing loss. A recent review of the Taiwan National Health Insurance Database of 553,286 TBI patients, compared to 1.1 million patients without TBI, demonstrated a 2.125x increased risk of hearing loss among the TBI patients (8). The most common source of trauma was a motor vehicle accident.

The inner ear as a source of dizziness and vertigo secondary to concussion has been increasingly recognized as we have developed better means for evaluating inner ear vestibular function. The inner ears are composed of 10 separate sensors (Anterior, Posterior and Horizontal semicircular canals (SCC) on each side; Utricle and Saccule on each side) that will each respond differently depending on the frequency and type of stimulus employed. Although we have seen great gains in our ability to evaluate each of these sensors in their spectra of responses, we are still just scratching the surface. The availability of testing for these sensors is limited in many clinical settings and most studies of post-concussion dizziness do not employ rigorous vestibular testing. Most studies evaluating PCD fail to include such objective measures of vestibular function and instead opt for physical exam measures and bedside balance tests. While these examinations are helpful, they lack the sensitivity/specificity and objectivity for discreet analysis of vestibular function. Consequently, the vestibular peripheral end organ is often ignored.

The studies that have looked at vestibular testing after concussion routinely find high rates of dysfunction. The most extensively evaluated has been the horizontal SCC. Horizontal SCC function is to provide vestibular input from horizontal rotations of the head. The most widely evaluated reflex has been the vestibulo-ocular reflex (VOR) of the Horizontal SCC—our means to prevent retinal slip from horizontal head movement. Studies using caloric testing (the extreme low frequency response range of Horizontal SCC VOR) in patients after head injury have demonstrated abnormalities ranging in 14–90% of patients (9–15). The frequency range for caloric testing is sub-physiologic and very few studies have looked at the higher, more physiologic range of frequencies. One that looked at dynamic visual acuity (frequencies within the physiologic range) found that 57% of children tested had abnormal results and 90% had some identifiable vestibular test abnormality (14). However, there were only 14% with an abnormal caloric test in that study.

The other SCCs (posterior and anterior), the Utricle and Saccule have been less well evaluated. Until recently, there has been limited means to evaluate the Superior and Posterior SCC. The advent of Video Head Impulse testing (vHIT) has allowed for the individual testing of each SCC. However, this takes place at a frequency above the physiologic frequency range. Similarly, testing of Utricular and Saccular function has been limited. Vestibular evoked myogenic potential testing has been recently employed among more centers. Unfortunately, this appears to be a relatively blunt tool in our arsenal, with results showing “present/not-present/hypersensitive” as the outcomes. Also, vestibular evoked myogenic potentials use sound as the stimulus which is unlike the more physiologic stimulus of movement or gravity on the Utricle or Saccule. Subjective Visual Vertical and more recently, the video Ocular Counter-roll test may provide a more quantitative and more physiologic stimulus to assess Utricular function going forward. Some proposed the Utricle and Saccule to be more predisposed to head injury than the SCCs, noting the importance of better means for evaluation of the Otolithic organs.

Tests of central vestibular dysfunction are frequently abnormal shortly after a concussion, but seem to normalize in most patients by 6 months after head injury (11, 16–18). While the assumption is that these tests correlate with the symptom of dizziness, the evidence for this is much less robust than for the peripheral vestibular system.

## Non-Vestibular Disorders

Not all dizziness is due to the vestibular system. The symptom “dizziness”, as we mentioned, is ill-defined. In some cases, patients complaining of dizziness are noting lightheadedness, unsteadiness/imbalance or other unusual, hard to describe feelings. There are some non-vestibular disorders the clinician should keep in mind with these vaguer symptoms.

In moderate and severe TBI patients, the incidence of pituitary dysfunction and subsequent pituitary hormone deficiency can be as high as 33–50% (19, 20). While the incidence among concussion patients is much lower, at close to 10%, the sheer number of concussion patients would imply a much greater number of post-concussion patients with pituitary dysfunction than seen among those with severe and moderate TBI. The clinician should keep in mind that the onset of symptoms may be gradual and delayed from the date of the head injury. In the post-concussion dizzy patient, endocrine evaluation should be considered if the patient fails to improve.

Autonomic dysfunction causing cardiovascular dysregulation can present with dizziness (21). Autonomic dysfunction among moderate and severe TBI patients has been most studied but it has been reported among mTBI patients as well. Most cases resolve without direct treatment, but there are some who have prolonged problems with dysautonomia. Sub-maximal exercise has been prescribed as a treatment for autonomic dysfunction in this setting.

## Central Vestibular Disorders

Among the central disorders, the most implicated is concussion itself. It has been reasonably assumed that DAI of the central vestibular pathways could affect the vestibular system causing dizziness. Oculomotor test abnormalities have been used as a proxy for central vestibular dysfunction. Tests suggestive of a central vestibular disturbance include abnormal results on pursuit tracking, saccade testing, optokinetic testing, gaze, near point convergence, and inability to suppress nystagmus (spontaneous nystagmus or induced by caloric testing). Complicating this picture is the use of medications that can affect the outcome of said oculomotor testing. Among studies looking at central vestibular test abnormalities in post-concussion dizziness, abnormal findings have been noted in 5–45% of patients. However, in the days after a concussion, the incidence of oculomotor abnormalities may approach 90%. The frequency of central findings appears to be lower beyond 6 months post-concussion, suggesting that much of this resolves spontaneously (15, 16). Oculomotor dysfunction, as noted above, may arise from DAI, but may also arise from direct injury to the oculomotor nerves (III, IV, and VI). These patients will often find relief with microprism lenses.

Other central processes can and do occur in the setting of post-concussion dizziness. Migraine can be a primary or a secondary disorder, although the presence of a post-concussion migraine may be a different entity from primary migraine diagnosis (2). Vestibular migraine, a variant of migraine with vestibular symptoms, is generally treated in the same manner. Intracranial hypertension, especially among obese patients and children, should be considered bearing in mind our growing obesity epidemic (22, 23). Nearly a third (29%) of secondary Normal Pressure Hydrocephalus (NPH) has been attributed to head trauma (24). The onset of symptoms is less typical, delayed from the time of the injury, but it seems to respond better to surgical intervention than idiopathic NPH. Chiari I malformation was seen in 8 of 427 children who underwent MRI for post-concussion symptoms (25). The clinical relevance in this study was uncertain, but one cannot exclude the exacerbation of a previously asymptomatic lesion such as this. However, Wan et al. (26) reported 12.9% of symptomatic Chiari presented shortly after mild head or neck injury.

## Fixed Peripheral Vestibular Disorders

A sudden loss of peripheral vestibular function will cause immediate onset of vertigo and dizziness. If this lesion is fixed and non-fluctuant, the patient will begin to develop central compensation almost immediately. The severity of the deficit will impact the duration of the symptoms with more severe deficits requiring longer durations for central compensation to occur. These patients often have symptoms beyond the first three weeks but should show significant improvement by 3 months post-injury. Labyrinthine concussion, most temporal bone fractures and cochleovestibular nerve traction injuries will present clinically in this manner (27). All will have varying degrees of vertigo, hearing loss, and tinnitus. The treatment is geared toward enhancement of central vestibular compensation. Elimination of vestibular suppressant medications and vestibular rehabilitation therapy will expedite this process. The outcome for vertigo and dizziness is quite favorable. However, these patients will generally have a quicker resolution of dizziness when vestibular rehabilitation exercises are employed. Hearing loss and tinnitus may demonstrate resolution or improvement, but long-term persistence of tinnitus and hearing loss is common.

## Fluctuating Peripheral Vestibular Disorders

The defining complaint of this group is the symptom of rotary vertigo spells. However, not all patients, and in particular post-head injury patients, can describe their symptoms well. Among the patients with persistent PCD beyond 3–6 months post-injury, this group will predominate. The most common fluctuating vestibular disorder by far is BPPV. Other disorders include dehiscence/fistula syndrome and delayed endolymphatic hydrops.

BPPV is by far the most common inner ear condition causing PCD. Among patients with concussions, the prevalence of BPPV ranges from 10 to 57% (9–15, 28). This is likely an underestimate of BPPV cases, due to either inadequate investigation or spontaneous resolution prior to evaluation. The classic symptoms of BPPV are positionally induced episodes of rotary vertigo that will have an irregular-irregular pattern of presentation that will confound the patient as to the source of the dizziness. This is due to the phenomenon of fatiguability found in BPPV—repetition of the provocative position will result in no vertigo/dizziness. The typical latency of the vertigo is 2–5 s after moving into the provocative position, and the duration is usually 15–30 s. The diagnosis is made during the Dix-Hallpike maneuver, demonstrating geotropic rotary nystagmus with the affected ear down.

Most cases of BPPV resolve spontaneously and will not require any treatment. For those that do not resolve spontaneously a variety of canalith repositioning maneuvers, of which the Epley maneuver is most common, are highly efficacious.

For BPPV, the most common type is unilateral posterior semicircular canalithiasis—free floating otoliths in the posterior semicircular canal in one ear. However, in PCD, we see a much higher incidence of atypical canal BPPV (horizontal and anterior semicircular canals), multiple canal involvement, bilateral BPPV and recurrent BPPV (28–31). The symptom presentation and the diagnostic findings on the Dix-Hallpike maneuver will be quite different for these variations and are more challenging to treat. In cases of PCD BPPV that do not resolve or persist, the possibility of BPPV secondary to another inner ear disorder (such as endolymphatic hydrops or dehiscence/fistula) should be considered. In this situation, without resolution of the underlying primary pathology, BPPV will persist/recur despite appropriate canalith repositioning maneuvers (32). It is important to note that BPPV does not cause hearing loss, tinnitus, or aural fullness. The presence of these symptoms should prod the clinician to look for additional diagnoses.

Labyrinthine dehiscence and perilymphatic fistula (LD/PLF) (33–38) seen in PCD will not generally respond to vestibular rehabilitation therapy and may be a source for recurrent BPPV. The hallmark symptoms for LD/PLF in PCD patients is strain-induced vertigo and dizziness. Some will also describe Tullio's phenomenon (sound-induced vertigo/dizziness). Associated symptoms include aural fullness, hearing loss (sometimes fluctuant), autophony, and tinnitus. Diagnostic testing may demonstrate abnormal results on certain provocative vestibular tests. Among these tests are cervical and ocular vestibular evoked myogenic potentials, platform pressure test, Nasal or Glottic Valsalva tests, Fistula (Hennebert) test and Tullio testing. For labyrinthine dehiscence patients, a high resolution (sub-mm slice thickness) CT scan will demonstrate absence of bone over part of the inner ear. The most common site for dehiscence is the superior semicircular canal but other sites have been implicated, including posterior semicircular canal dehiscence, cochlear-facial dehiscence, carotid-cochlear dehiscence, and internal auditory canal-cochlear dehiscence. A non-displaced temporal bone fracture can present with a LD/PLF syndrome (36). Also note, a round window or oval window perilymph fistula will have a normal CT scan and present in the identical manner as described above (37).

Initial treatment for LD/PLF includes elimination of straining (or provocative stimulation) which may include a limited duration of bedrest. Carbonic anhydrase inhibitors and diuretics have been utilized with success in some patients as well. For those who have persistent symptoms despite such medical measures, surgical repair may be warranted.

Endolymphatic hydrops of the cochlea and the vestibular system has been demonstrated in numerous post-concussive temporal bone specimens. The term “secondary endolymphatic hydrops” (SELH) is used, since this is felt to be one of the later/chronic developments in the pathology of labyrinthine concussion (39, 40). Clinically, these patients may have worsening of hearing and vertigo weeks or months after concussion, mimicking Meniere's disease. These PCD patients will also not respond to vestibular rehabilitation therapy due to the fluctuant nature of the disorder. Vestibular testing will often demonstrate evidence of peripheral vestibular system dysfunction. Hearing loss will frequently demonstrate low frequency fluctuant hearing loss and electrocochleography will often be abnormal. One of the clinical differences between SELH and LD/PLF is the timing of onset of symptoms. Whereas, both can show progression of hearing loss and vertigo/dizziness, LD/PLF usually has early onset. SELH presents weeks to months after the head injury.

Treatment of SELH is essentially the same as primary Meniere's disease. Conservative medical therapy is geared toward dietary changes, diuretics, and symptomatic care of acute vestibular episodes. Surgical treatment is reserved for those who have intractable vertigo despite maximal medical management.

## Management

### Less Than 3 Weeks Post-concussion

An estimated 85–90% of concussion patients will have resolution of their symptoms within days to weeks of the injury. Consequently, these “early resolvers” will not undergo testing and have no need to undergo testing. While there is no proven treatment, the recommendation for brain and body rest seems reasonable to prevent exacerbation of a labyrinthine dehiscence/fistula and prevent a second concussion while the patient is recovering. Once acute complications have been excluded, this group should be given supportive care. If post-concussion dizziness persists beyond 3 weeks, more active treatment should begin.

### Three Weeks to 3 Months Post-concussion

The most common disorder causing PCD that persists beyond 3 weeks is BPPV. Appropriate treatment with canalith repositioning will provide significant benefit to this group of patients. Fixed peripheral vestibular disorders and central vestibular dysfunction will show improvement during this period. Vestibular rehabilitation therapy during this period should help advance central vestibular compensation. Submaximal exercise therapy appears to have some benefit in resolving autonomic dysfunction. This is usually all that is necessary for PCD patients during the 3 weeks to 3 months post-concussive period. During this time, even patients with severe fixed peripheral vestibular deficits should demonstrate improvement in PCD. The more severe vestibular loss patients may continue to improve beyond this point, and continued vestibular rehabilitation exercises may be warranted for this group. However, patients with discreet episodes of prolonged rotary vertigo or significant PCD beyond 3 months, deserve further attention and likely will require medical and/or surgical intervention.

In a prospective study of 58 mTBI patients with dizziness, evaluated within 1–3 days post-injury, Hoffer et al. (41) demonstrated efficacy of vestibular rehabilitation therapy. The post-traumatic positional vertigo group (28% of patients) had no objective vestibular findings except for BPPV. The average period for return to work and symptom remission was less than a week. The rest of the patients had objective evidence for VOR abnormalities, and they were divided into two groups—post-traumatic migraine associated dizziness (41%) and post-traumatic spatial disorientation (19%). After 6–8 weeks of vestibular rehabilitation therapy, 84% of the migraine group and 27% of the spatial disorientation group had significant symptom improvement and improvement on objective VOR testing. Average time to symptom resolution was 7.9 weeks for the migraine group and 39 weeks for the spatial disorientation group. Only two patients (3.4%)—both in the spatial disorientation group—were still symptomatic at the time of the study, beyond 1 year.

### Three Months Post-concussion

By 3 months post-concussion, patients with fixed peripheral vestibular deficits and central vestibular dysfunction should be significantly improved or improving with vestibular rehabilitation therapy. BPPV should have resolved spontaneously or been appropriately treated. Those who have persistent or worsening problems with dizziness deserve a more aggressive evaluation. MRI, if not already done, should be performed to exclude central processes discussed above such a Chiari malformation, normal pressure hydrocephalus and other coincidental pathologies unrelated to the concussion. Assessment of intracranial pressure elevation should be considered in the PCD patient who has constant pressure headaches. A high-resolution CT scan of the temporal bones should be performed to evaluate the integrity of the labyrinthine bone for fractures, dehiscences and other anomalies. If there is suspicion of pituitary dysfunction, the patient should be referred for endocrine evaluation.

A full audiometric evaluation should be completed, including pure tone and speech audiometry, impedance testing, and electrocochleography. The presence of hearing loss, especially asymmetric hearing loss, raises the possibility of inner ear vestibular dysfunction. Low frequency losses are more consistent with SELH, especially if it is delayed and there is documentation of fluctuation. Low frequency loss with a conductive component is more suggestive of LD/PLF. High frequency sensorineural losses, sloping or notched at 3–8 kHz, suggest labyrinthine concussion.

A full vestibular evaluation should be completed including assessment of oculomotor function (Saccades, OPK, Pursuit, Gaze), semicircular canal function (caloric testing, vHIT, rotational studies), otolithic function (cVEMP, oVEMP, SVV/H, Ocular Counter-roll test) and posture (computerized dynamic platform posturography). Provocative tests for specific pathology should be performed including looking for evidence of labyrinthine dehiscence or perilymphatic fistula (Platform Pressure Test, VNG fistula Test, Tullio test, Nasal and Glottic Valsalva test) and test for BPPV (Dix-Hallpike, Lateral Roll, Multi-axial positional chair). Rotational studies (both rotary chair and active head rotation testing) will elucidate on the state of central compensation. The more testing performed, the more data and the better to identify the etiology of PCD in each individual patient.

The purpose of the extensive evaluation is to direct treatment to the specific pathology. The pathologies identified will tend to be those of the fluctuating peripheral vestibular type, along with a handful of other pathologies. In general, treatment will consist of non-medical measures, medication, and surgical intervention. Most patients will do well with non-medical and medical therapy. A smaller portion will require surgery to stabilize their fluctuating lesion. Once the fluctuating vestibular disease is stabilized, vestibular rehabilitation therapy is instituted, and should show significant benefit within 6–12 weeks.

## Otolithic Dysfunction

We have only recently begun clinical evaluation of otolithic function, but many have suggested that the otolithic organs (Utricle and Saccule) are the most injured vestibular sense organs (42–45). The most prominent evidence of otolithic damage post-concussion is indirect. The most common cause for PCD is BPPV and the pathophysiology of BPPV is dislocation of otoconia from the Utricle. This can only happen with direct damage to the Utricle. Currently, we can only speculate what are the implications of the dislodgement of otoconia from the Utricle, but as objective Utricular testing improves and becomes more widespread, we will learn. The Saccule has been implicated to be more commonly injured than the Utricle but due to intralabyrinthine anatomy, Saccular otoconia would not easily transmit to the semicircular canals and cause BPPV.

Symptoms from otolithic dysfunction are quite different from the symptoms of semicircular canal dysfunction (42). While semicircular canal stimulation will provoke nystagmus and the symptom of rotary vertigo, the symptoms of otolithic dysfunction are more subtle. The VOR from the Utricle results in ocular torsion and asymmetric vertical stationary movements of the eye. Unilateral abnormal Utricular stimulation can result in blurred vision or diplopia that can often be augmented with head position. Utricular dysfunction can be identified on oVEMP, SVV testing and Ocular Counter-roll testing. Indirectly, some of these patients can be identified by their response to microprism lenses. Saccular reflexes are primarily to the postural muscles via the vestibulospinal reflex. Saccular dysfunction often results in poor balance or the sensation of rocking. Abnormal saccular responses can sometimes be identified on cVEMP and computerized dynamic post-urography.

## Malingering

Many cases of trauma resulting in concussion also result in litigation. When litigation is involved, secondary gain incentives may obscure the clinical picture. The clinician must always keep the specter of malingering in mind. Non-physiologic test results and non-compliance with testing and treatment should arouse suspicion.

The DSM V criteria for malingering include any two of the following:

- Medical-legal context of presentation.
- Marked discrepancy between the individual's claimed stress or disability and objective findings and observations.
- Lack of compliance during diagnostic evaluation and prescribed treatment regimen.
- Antisocial personality disorder.

While the diagnosis of malingering can be made with the above criteria, rather than making that diagnosis we prefer to comment on whether the patient meets the DSM V criteria for malingering or not. This leaves the final judgment to the courts over whether the patient is malingering.

When evaluating a patient suspected of malingering, one must consider that despite non-physiologic results, the patient may also have pathology. In a study among dizzy patients with pending litigation (46) ~25% had pathologic findings, and 25% had non-physiologic findings on vestibular testing. The other 50% had both pathologic findings and non-physiologic findings on testing. Looking at this data one could say that 75% of the group were malingering/exaggerating or one could say that 75% had pathology. Both statements are valid. Objective vestibular testing is required to determine vestibular damage.

## Combinations and Bilaterality

Osler's rule that symptoms should be explained by one neurologic lesion does not apply very well in PCD. While the literature is relatively silent on this issue, our experience in dealing with PCD patients is that they frequently have more than one pathologic process ongoing. It may just be the tertiary referral nature of our practice, but combinations of differing pathologies seem to be the rule, rather than the exception. Especially early after concussion, a mixture of central dysfunction (defined by ocular-motor abnormalities) and associated BPPV is a common combination. In fact, BPPV is seen with a host of other PCD disorders including labyrinthine concussion, temporal bone fracture, LD/PLF and SELH. In our clinic, where most of the patients are several years out from injury and have been unsuccessfully treated elsewhere, we usually see a mixture of peripheral dysfunction and central dysfunction. The important point to the practicing clinician is that even after identifying a cause for PCD, you must consider the possibility of a concomitant disorder contributing to PCD. Further, bilaterality of inner ear damage is quite common, especially BPPV and labyrinthine concussion.

## Conclusion

Dizziness is common after concussion. Most cases (85–90%) will resolve in the first 3 weeks without treatment. If not resolved within 3 weeks, assessment for BPPV with canalith repositioning should be performed. Otherwise, the patient should begin vestibular rehabilitation therapy. Three months post-concussion most cases of central vestibular dysfunction and fixed peripheral vestibular disorders will be either resolved or greatly improved. For patients who are still significantly symptomatic after 3 months, a more extensive evaluation is warranted. The majority of those who have persistent problems will be those who have fluctuating vestibular disorders and a handful of other disorders. A thorough audio-vestibular test battery should be performed, and disease-specific therapy should be instituted for these patients. Using this format, we can eliminate unnecessary evaluation for most patients who will have resolution, and direct resources to those who need in-depth evaluation and treatment.

## Author Contributions

The author confirms being the sole contributor of this work and has approved it for publication.

## Conflict of Interest

The author declares that the research was conducted in the absence of any commercial or financial relationships that could be construed as a potential conflict of interest.

## Publisher's Note

All claims expressed in this article are solely those of the authors and do not necessarily represent those of their affiliated organizations, or those of the publisher, the editors and the reviewers. Any product that may be evaluated in this article, or claim that may be made by its manufacturer, is not guaranteed or endorsed by the publisher.
